# Age, period and cohort analysis of age-specific maternal mortality trend in Ethiopia: A secondary analysis

**DOI:** 10.1371/journal.pone.0224220

**Published:** 2020-01-16

**Authors:** Biniam Getachew, Tippawan Liabsuetrakul, Shama Virani, Yirgu Gebrehiwot

**Affiliations:** 1 Epidemiology Unit, Faculty of Medicine, Prince of Songkla University, Hat Yai, Songkhla, Thailand; 2 Department of Environmental Health Sciences, University of Michigan, Ann Arbor, MI, United States of America; 3 Obstetrics and Gynecology Department, Addis Ababa University, Addis Ababa, Ethiopia; Aga Khan University, PAKISTAN

## Abstract

**Background:**

Maternal mortality (MM) was persistently high for several decades in Ethiopia though it has declined in recent years. The roles of time-varying elements in this decrease are unknown. Analyzing MM with age-period-cohort analysis will provide evidence to policymakers to re-direct resources towards vulnerable age groups. The aim of this analysis was to determine the role of age effect, period effect and birth cohort effect on the trend of age-specific maternal mortality in Ethiopia.

**Methods:**

Age-period-cohort (APC) analysis was applied to examine the effect of age, period and birth cohort on MM in Ethiopia using data from the Ethiopian Demographic and Health Survey (EDHS) from years 2000, 2005, 2011 and 2016. Age-specific maternal mortality rates were calculated using standardized maternal death compared to age-specific population per 100,000 woman-years of exposure and the trend was analyzed.

**Result:**

In most age groups, the MM rate decreased in 2015 compared with the previous years except for older women. According to the APC analysis, the age-cohort effect explains the MM rate better than age-period effect. The period effect shows the risk ratio of MM after 2005 decreased compared with before. The cohort effect illustrates women born after 1980 has lower risk ratio compared with the older one.

**Conclusion:**

Maternal mortality in Ethiopia declined overall in recent years. However, certain age groups still face high maternal mortality rates. A national policy on MM reduction interventions for the identified high-risk age groups is required.

## Background

Maternal death is the death of a woman while pregnant or within 42 days of termination of pregnancy, irrespective of the duration and site of the pregnancy, from any cause related to or aggravated by the pregnancy or its management but not from accidental or incidental causes [1]. Globally, maternal mortality ratio declined by 44% from 385 maternal deaths per 100,000 live births in 1990 to 216 in 2015, yielding an average annual decline of 2.3%. Yet Millennium Development Goal 5, which was to reduce maternal mortality ratio by three quarters between 1990 to 2015, was not achieved in many developing countries [2]. Although the trend of maternal death has been continuously declining by time, 99% of maternal deaths still occurred in developing countries. Maternal deaths in sub-Saharan Africa region accounted for 62% of those in developing countries [3]. Maternal mortality is an important global health issue (indicator) and is included in the targets of Millennium Development Goal (MDG) and Sustainable Development Goal (SDG) [4].

In Ethiopia, a study revealed persistently high maternal mortality for several decades [5]. This was corroborated by the Ethiopian Demographic and Health Surveys (EDHS) in 2000, 2005, and 2011 [6–8], which report similarly high maternal mortality. The main four cause of maternal mortality in Ethiopia in the last decade were obstructed labor/uterine rupture, hemorrhage, hypertensive disorders of pregnancy and sepsis/infection [9]. However, maternal mortality decreased by 2016 according to EDHS [10]. The World Health Organization 2015 estimation of maternal mortality for Ethiopia [3] was even lower than EDHS 2016.

It is likely that the decrease in maternal mortality may be attributed to targeted interventions. The Government of Ethiopia implemented several different interventions to reduce maternal mortality which included expansion of health facilities, training of a large number of the health workforce, wide implementation of health extension programs, expansion of family planning service, implementation of maternity waiting homes for women who live far from health facility and adaptation of liberal abortion law to mention some [11–13] With all the above-mentioned efforts, maternal mortality trend in Ethiopia has shown a reduction by 71.8% from 1990 to 2015 which was a little bit lower than MDG 5 target 6 to achieve reduction of maternal mortality by three-quarters [3].

Most of the studies failed to report analysis using age-specific maternal mortality trend and to see the period and cohort effect of maternal mortality through years. It is necessary to describe how time-varying elements such as the age, period and birth cohort effects contribute to trends over time [14]. According to the analysis of age effect, maternal mortality rate shows variation across different age groups [15–17]. A period effect looks at a population (regardless of age) and sees if a particular time frame prior to diagnosis (occurrence of the event) may be important, such as economic development, expansion of health service, fertility trends and epidemics like HIV/AIDS. Birth cohort effect considers the generational effect as exposures might occur around time of birth [18, 19].

Currently, there is a paucity of published studies that focus on the trend of maternal mortality using APC analysis, particularly for Ethiopia. This study thus aimed to determine the role of age effect, period effect and birth cohort effect on the trend of age-specific maternal mortality in Ethiopia. This analysis provides evidence of which age groups are most at risk of maternal mortality and it will be useful to inform policymakers on how to re-direct resources and provide greater effectiveness towards the most vulnerable age groups.

## Methods

### Study design and data sources

A secondary analysis using data from the Ethiopian Demographic and Health Surveys (EDHS) of 2000, 2005, 2011 and 2016, which is publicly available, was carried out.[6–8, 10] Even though, EDHS 2011 was reported in 2011, the data was collected in 2010; so the data from EDHS 2011 directly used as 2010. Maternal mortality rate was calculated using maternal death compared to age-specific population per 100,000 woman-years of exposure. In this analysis, maternal mortality rate is used rather than ratio because the data from the surveys have population data rather than number of live births data. Moreover, it is advised to use rate than ratio while analyzing age-specific maternal mortality due to maternal mortality rate yields more accurate estimates of the lifetime risk of maternal death than ratio [16].

To generate data for consecutive years, data interpolation for 2015 based on the available data using smoothing spline method of generalized additive models was applied as mentioned in Holford 2006 [18]. Direct standardization of the rate was done to 2015 which makes the analysis methodologically sounds well and easier to compare among different periods and cohort. Data were arranged in 5-year maternal age (A) grouping from 15 to 49 years of old and coded 15–19 as 15, 20–24 as 20, 25–29 as 25, 30–34 as 30, 35–39 as 35, 40–44 as 40, and 45–49 as 45. Whereas, the period (P) from 1996 to 2015 was categorized and coded in 5-year interval as follows; 1996–2000 was coded as 2000, 2001–2005 as 2005, 2006–2010 as 2010 and 2011–2015 as 2015. The birth cohort (C) was obtained by subtracting the age (A) from the period (P) (C = P-A). Thus, the cohort ranges from 1951 to 2000 was grouped and coded as follows: 1951–1955 as 1955, 1956–1960 as 1960, 1961–1965 as 1965, 1966–1970 as 1970, 1971–1975 as 1975, 1976–1980 as 1980, 1981–1985 as 1985, 1986–1990 as 1990, 1991–1995 as 1995, and 1996–2000 as 2000.

### Statistical analysis

The age-period-cohort (APC) model represents a classic epidemiological approach that can be used to extract information from cross-sectional data regarding historical changes in mortality and morbidity risk. The APC model is categorized into multiple regression models and can be expressed as:

Y=μ+αX1+βX2+γX3+ε;

where X1, X2, X3 represent age, period, cohort, respectively, and α, β, *γ* represent their parameter estimates, respectively. When referring to the estimation of maternal mortality data, the model can be written as a log-linear Poisson model:

ϕ(λijk)=μ+αi+πj+γk+ϵij

where *ϕ*(…) is the log link function, *λijk* denotes maternal death rate, *μ* represents the intercept, *αi* denotes the *i*^th^ row age effect or the coefficient for the *i*^th^ age group, *πj* denotes the *j*^th^ column period effect or the coefficient for the *j*^th^ time period, *γk* denotes the *k*^th^ diagonal cohort effect or the coefficient for the *k*^th^ cohort; ε*ij* denotes the random error with expectation E(ε*ij*) = 0. For the age group, we employ the index *i* (= 1,…,*I*), for period *j* (= 1,…,*J*) and for birth cohort *k* = *j*-*i*+*I* (= 1,…,*K = I+J*-1).

The known problem related to the APC analysis is the linear dependence of the three time indices (age, period and birth cohort) and is called the non-identification problem.[19] To deal with this non-identification problem, two models are run. In the first model, the age-cohort-period (AC-P) model, age and cohort effects are fit to the data with the period effects constrained to be 0 on average with 0 slope. In the second model, the age-period-cohort (AP-C) model, age and period effects are fit to the data with the cohort effects constrained to be 0 on average with 0 slope [14]. Due to the highest number of maternal mortality rate, age-group 30–34 and 1980 birth cohort were considered to be referent group whereas the period 2005 was used as referent group considering the implementation of the liberal abortion law in the country during that period. The effect of age, period and cohort was analyzed using APC and ACP model.

For data management and analysis R version 3.3.3 (R Foundation for Statistical Computing, Vienna, Austria) using gam, apc, and Epi package was used [20]. Fitted deviance was used to evaluate the model. This analysis was approved by the Ethics Review Committee of Faculty of Medicine, Prince of Songkla University, Thailand (EC no. 60-253-18-5).

## Results

A total of 819 maternal deaths from the four surveys included in this study. Table 1 shows age-specific maternal mortality rate (asMMRate) in different years. Within each period, asMMRate peaked for women ages 30–34 years, although from 2001–2005 women ages 35–39 years had the highest asMMRate.

**Table 1:** Age-specific maternal mortality rate across different periods.

Age	Period			
	1996–2000	2001–2005	2006–2010	2011–2015
15–19	94.27	47.12	52.68	41.58
20–24	185.27	137.70	97.64	70.10
25–29	194.92	186.56	103.03	71.39
30–34	256.54	197.60	260.01	145.60
35–39	193.93	218.79	154.15	74.58
40–44	108.33	41.66	141.67	91.67
45–49	43.98	14.44	73.31	73.31

In this analysis, the effect of period and cohort was evaluated separately as age-period-cohort (AP-C) and age-cohort-period (AC-P) model. Table 2 shows the effect of age-cohort, age-period-cohort and age-period analysis. Based on the low residual deviance, the age-period-cohort is the best model to explain the effect of maternal mortality. However, to identify which effect is most important, we focus on the effects of cohort and period separately. Age-cohort showed lower residual deviance than age-period indicating the age-cohort model fits the data better than the age-period model. The cohort effect contributes more to the trend than period effect.

**Table 2:** Residual deviance model for AP,AC, and APC to age only model.

Model	Resid. Df	Resid. Dev.	Df	Deviance	p value
Age	24	115.356			
Age-drift	23	56.47	1	58.89	<0.0001***
Age-Cohort	20	40.74	3	15.73	0.0013 **
Age-Period-Cohort	18	37.74	2	2.99	0.2236
Age-Period	21	51.71	-3	-13.97	0.00295**

AC: age-cohort; AP: age-period; APC: age-period-cohort; Resid. Dev: residual deviance; Df: degree of freedom

** significant at 0.01

*** significant at 0.001

**Fig 1:**
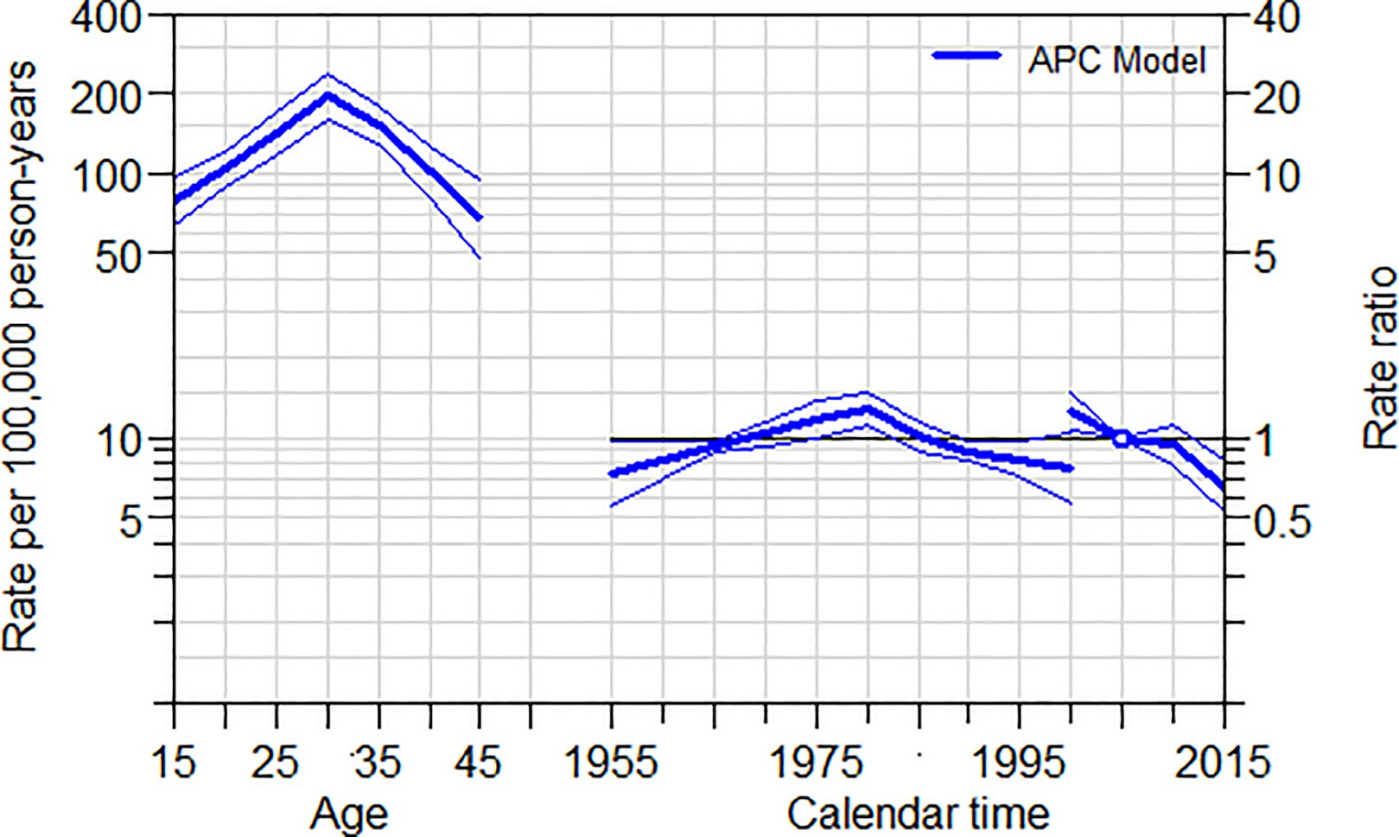
Age-period-cohort model trend analysis of maternal mortality.

**Fig 2:**
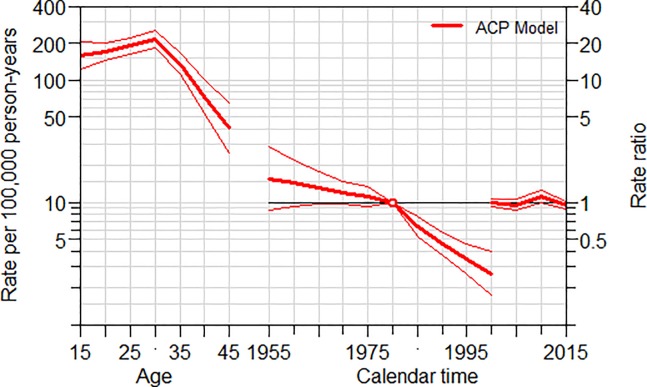
Age cohort period model trend analysis of maternal mortality.

In order to see the period effect (Fig 1), the AP-C model constrains the cohort effects to be 0 (center). As shown in Fig 1, the age (left) shows a peak with a maximum maternal mortality rate recorded at the age of 30–34. The period effect (right) shows a decreasing risk of maternal mortality compared to the reference year of 2005 in recent years. On the other hand, to evaluate the cohort effect (Fig 2), the AC-P model constrains the period effects to be 0 (right). The age effect (left) peaks at 30–34 age with a maternal mortality rate of 213.8 per 100,000 woman-years of exposure. As cohort effect using the reference year 1980, the younger generation has a lower risk of maternal mortality compared to older generations when compared to women born in 1980.

## Discussion

Age-specific maternal mortality trend declined across age groups in each study period. Women ages 30–34 years had high maternal mortality across the study periods compared to all other age groups. The cohort effect showed that younger generations had lower risk ratios of maternal death than older generations. The period effect showed that the risk of a mother to die due to pregnancy and its complication decreased in recent years when compared to the risk in 2005.

Globally, it was reported that maternal mortality decreased significantly in almost all age groups from 1990 to 2015 and that maternal mortality increased with age [21]. Although maternal mortality decreased overall in Ethiopia in recent years [5, 10], we found that maternal mortality increases with age till the age of 30–34 and then decreases in older women. The discrepancy between the global statistic and our findings may be due to the fact that the global study included data from developed and developing countries, and may not be representative of Ethiopia alone. Another possible explanation might be because women aged 30–34 years in Ethiopia had high births or risks as supported by a previous study which aggregated results from the Demographic and Health Survey (DHS) from 38 countries revealing that maternal mortality rates were higher where the risk per birth is high and/or where more births per women happen [22].

Consistently high maternal mortality in the age group 30–34 years throughout different periods in our study was not accordance with the previous studies in Ethiopia [17], Malawi [23], and Bangladesh [16] which explained age groups of 25–29 years have a higher number of maternal deaths. This difference may be due to different data sources used. We used the Ethiopian DHS, while the previous study used the Global Burden of Disease (GBD) 2013. Although the Ethiopian Demographic and Health Survey 2016 reported the highest total fertility rate in women aged 25–29 years [10] and more fertile women had a higher risk of maternal death due to pregnancy related complication [24], we could not find the highest mortality in this age group.

Our findings from the age-period-cohort analysis showed that younger generations had lower maternal mortality risk than the older ones–which was the cohort effect. This is likely due to the high effort of the Ministry of Health and its partners in reducing maternal mortality through policies like adaptation of liberal abortion law since 2005. For example, a woman who joined the reproductive age group after adaptation of liberal abortion law might face the minimal lifetime risk of maternal death due to abortion. This was supported by different studies stating maternal death due to abortion declined significantly in the last decade in Ethiopia [9]. As it is almost impossible to separate the time effect, the adaptation of liberal abortion law could be seen in both cohort and period effect.

The period effect of decreasing maternal mortality would be explained by the improvement of most maternal health indicators and health reforms in Ethiopia such as modern contraceptive use increasing from 6% in 2000 to 35% in 2016, or delivery by skilled birth attendance increasing from 6% in 2000 to 28% in 2016 [10, 11] Moreover, the Health Extension Program which consists family planning services, ANC, immunization and activities that facilitate pregnant women to give birth at health institutions through reaching underserved women in rural area since 2003 would have played a great role in maternal health improvement [25]. The government of Ethiopia has implemented the healthcare financing reforms in 2005 stating payment exempt for prenatal, delivery, postnatal and family planning services provided by primary healthcare units [26]. In addition, 24-hour four-wheel drive ambulances in every rural district with mobile telephone network have been established since 2012 [27].

The findings of this study are useful because there is very little evidence in the literature on age-specific maternal mortality trends in low-income countries including Ethiopia. Age-specific maternal mortality, as used in this approach, is helpful for policymakers to identify vulnerable age groups to set up the priority for the country where is limited-resource settings as the maternal mortality rate or ratio was not constant throughout all ages [16].

There are some limitations to this study. First, the data used in this analysis was aggregated data, which individual analysis was limited. Second, to do APC analysis with 5-year age and period interval, data interpolation was done for the year 2015 which might be somehow different from the real one. Finally, the reasons behind why women aged 30–34 years had the highest maternal mortality could not be determined.

## Conclusion

Age-specific maternal mortality in Ethiopia declined overall in recent years. Not only the age-period effect but also the age-cohort effect was important to consider. However, certain age groups still face high maternal mortality rates. Because of this, the Ministry of Health (Ethiopia) should consider maternal mortality reduction interventions with respect to high-risk age groups. Recent policies introduced by the Ministry of Health and its partners might have contributed to the lower risk of maternal mortality in younger generations and in recent years described here.

## Abbreviation

AC: Age cohort
ACP: Age cohort period
ANC: Antenatal care
AP: Age period
APC: Age period cohort
asMMRate: Age-specific maternal mortality rate
DHS: Demographic and Health Survey
EDHS: Ethiopian Demographic and Health Survey
MDG: Millennium Development Goal
MM: Maternal mortality
MMR: Maternal mortality ratio
